# Chemical Kinetic Model of Multicomponent Gasoline Surrogate Fuel with Nitric Oxide in HCCI Combustion

**DOI:** 10.3390/molecules25102273

**Published:** 2020-05-12

**Authors:** Chao Yang, Zhaolei Zheng

**Affiliations:** Key Laboratory of Low-grade Energy Utilization Technologies and Systems, Ministry of Education, Chongqing University, Chongqing 400044, China; yangchauo@foxmail.com

**Keywords:** simplified mechanism, HCCI, NO, diisobutylene, ignition delay time

## Abstract

This study presents a simplified mechanism of a five-component gasoline surrogate fuel (TDRF–NO) that includes *n*-heptane, isooctane, toluene, diisobutylene (DIB) and nitric oxide (NO). The mechanism consists of 119 species and 266 reactions and involves TDRF and NO submechanisms. Satisfactory results were obtained in simulating HCCI combustion in engines. The TDRF submechanism is based on the simplified mechanism of toluene reference fuel (TRF) and adds DIB to form quaternary surrogate fuel for gasoline. A simplified NO submechanism containing 33 reactions was added to the simplified mechanism of TDRF, considering the effect of active molecular NO on the combustion of gasoline fuel. The ignition delay data of the shock tube under different pressure and temperature conditions verified the validity of the model. Model verification results showed that the ignition delay time predicted by the simplified mechanism and its submechanics were consistent with the experimental data. The addition of NO caused the ignition delay time of the mechanism simulation to advance with increasing concentration of NO added. The established simplified mechanism effectively predicted the actual combustion and ignition of gasoline.

## 1. Introduction

Compared with traditional compression ignition (CI) and spark ignition (SI) engines, homogeneous charge compression ignition (HCCI) engines have become a focus of research among internal combustion engines because of their efficient and clean combustion methods. However, combustion of HCCI is sensitive, and slight changes in the mixture state in the cylinder greatly affect the combustion [1]. In addition, HCCI has problems, such as a narrow range of operating conditions, difficulty in catching fire and high HC and CO emissions in HCCI combustion.

To solve these problems, scholars have adopted various advanced technologies such as intake air heating [2] and increasing the compression ratio of internal combustion engines [3], control strategy with dual fuel [4] and simulation of surrogate fuels [5]. The use of numerical simulation with chemical kinetics as the core is one of the most appropriate means to explore combustion mechanism and help achieve accurate control of HCCI.

Gasoline is a complex mixture that contains hundreds of hydrocarbons, including olefins, naphthenes, alkanes and aromatics. The study of the chemical kinetic mechanism of gasoline is complicated. Increasing the number of components will exponentially increase the reaction, material and thermophysical parameters; hundreds of components in gasoline will complicate the reaction mechanism to an unacceptable level [6]. Therefore, using one or several components to describe the physicochemical properties of gasoline fuel has become a feasible research trend.

Isooctane is the first considered surrogate fuel for research on selecting gasoline substitute mixture components. Isooctane has a high-octane number and is widely used in gasoline HCCI combustion simulations [7]. Galmiche et al. [8] and Liu et al. [9] constructed a simplified kinetic model for HCCI combustion of isooctane to control the ignition delay time of fuel in the shock tube and predict the heat rate and fuel consumption. The octane number of isooctane is fixed (RON = MON = 100) and thus cannot reflect the important characteristic of gasoline octane change.

The cetane number of *n*-heptane is similar to that of diesel oil [8] and can be used to match the octane number of gasoline fuel by mixing different ratios of *n*-heptane and isooctane. The mixture is called primary reference fuel (PRF). Scholars have conducted much research on the kinetic model of PRF chemical reaction [10,11,12]. The experimental results of Risberg et al. [13] showed that a large difference exists in flame stability between PRF and gasoline; moreover, the laminar flame propagation speed of PRF is higher than that of standard gasoline. To better simulate the combustion of gasoline HCCI, researchers have proposed a mixture of three materials, namely, isooctane, *n*-heptane and toluene, as surrogate fuel (toluene reference fuel (TRF)) for gasoline [14,15,16].

Andrae et al. [14] added the PRF skeletal mechanism to the detailed toluene mechanism and obtained a TRF mechanism containing 1121 species and 4961 reactions. The prediction results of this mechanism are consistent with the experimental results of ignition delay time in shock tube, fast compressor and HCCI engine. Zhang et al. [15] proposed a simplified TRF mechanism with 70 components and 196 reactions for HCCI conditions. Gasoline three-component surrogate fuel containing *n*-heptane, isooctane, and toluene was used in shock tubes. The ignition delay time was also verified. Coskun et al. [16] used the TRF chemical kinetic mechanism consisting of 137 species and 633 reactions to study the effects of various initial conditions and fuel/air equivalent ratios (*ϕ*) on the combustion and emission characteristics of HCCI engines.

Considering the representative components of olefins in actual gasoline, many scholars added diisobutylene (DIB) as a representative of olefins to TRF for forming a quaternary alternative fuel for gasoline. This quaternary fuel is referred to as TDRF. Fikri et al. [17] conducted shock tube experiments with multiple groups of gasoline substitute mixtures of TDRF fuel under different mixing conditions. The results showed that the ignition delay of the quaternary TDRF fuel (20% *n*-heptane/25% isooctane/45% toluene/10% diisobutene by liquid volume) was closest to actual gasoline. The experimental data of the ignition delay time in shock tube can be used to verify the mechanism of the quaternary gasoline surrogate mixture.

HCCI combustion is a lean, low-temperature combustion process controlled by the fuel’s chemical reaction kinetics, which plays a vital role in the entire combustion process and emission. As important components of the burned exhaust gas, NO exhibits active chemical properties that affect HCCI combustion.

The interaction between NO and small-molecular hydrocarbon fuels was first studied [18,19]. Frassoldati et al. [19] simulated the interaction between NO and hydrocarbon fuels at high temperature by using chemical kinetics and obtained NO cross reaction with C1–C4 fuel. Gasoline contains a large number of macromolecular hydrocarbons, so the interaction between NO and macroalkanes is also discussed in detail [20,21,22,23].

Wang et al. [20] experimentally investigated the effect of NO on the HCCI combustion of *n*-heptane and isooctane. Contino et al. [23] used a PRF-NO chemical reaction kinetic model to analyze the effect of NO on isooctane oxidation. Zheng et al. [24] constructed a simplified mechanism of TRF–NO containing 80 species and 184 reactions; they compared the simulated and experimental values of TRF–NO under different operating conditions and found that the simplified mechanism of TRF–NO can better reflect the tendency of the ignition delay time of HCCI engines to change with the concentration of NO added.

Few studies are available on the chemical kinetic mechanism of TDRF–NO. Exploring the TDRF–NO mechanism can increase understanding of the HCCI combustion mechanism of gasoline engines and has profound significance for studying the relationship between gasoline substitutes and NO.

In this study, NO and TDRF (i.e., *n*-heptane, isooctane, toluene and diisobutylene) were selected as research objects. First, a DIB simplification mechanism was added based on the predecessor TRF simplification mechanism. Temperature sensitivity and reaction path were analyzed to determine key reactions and influence of NO on DIB. The reaction mechanism of TDRF–NO was constructed and the calculation results were compared with experimental values under different working conditions to verify the effectiveness of the construction mechanism.

## 2. Results and Discussion

The mechanism of TDRF–NO includes two parts, TDRF and NO submechanisms. Surrogate fuel TDRF (i.e., *n*-heptane, isooctane, toluene and diisobutylene) can better simulate the ignition characteristics of gasoline, and addition of NO can make the ignition delay time advance with the increase in concentration. In our previous research, a simplified TRF mechanism was constructed, and the mechanism was verified under shock tube conditions [25]. We added the DIB and NO submechanisms. The verification results showed that the model and experimental data had a good consistency in ignition delay time. Moreover, the ignition delay time of the model agreed well with the experimental data.

### 2.1. DIB Submechanism

DIB is a mixture of two conjugated olefins, 2,4,4-trimethyl-1-pentene (JC_8_H_16_) and 2,4,4-trimethyl-2-pentene (IC_8_H_16_). The chemical kinetic mechanism of some TDRF fuels only considers JC_8_H_16_. Basing on the detailed mechanism of DIB, we simplified the IC_8_H_16_ mechanism in the detailed mechanism of DIB by using two methods, reaction rate and temperature sensitivity analyses. The simplified chemical kinetic model consists 37 species and 34 reactions and is combined with the existing simplified chemical kinetic model of JC_8_H_16_ [26] to construct a complete simplified DIB model.

**Figure 1:**
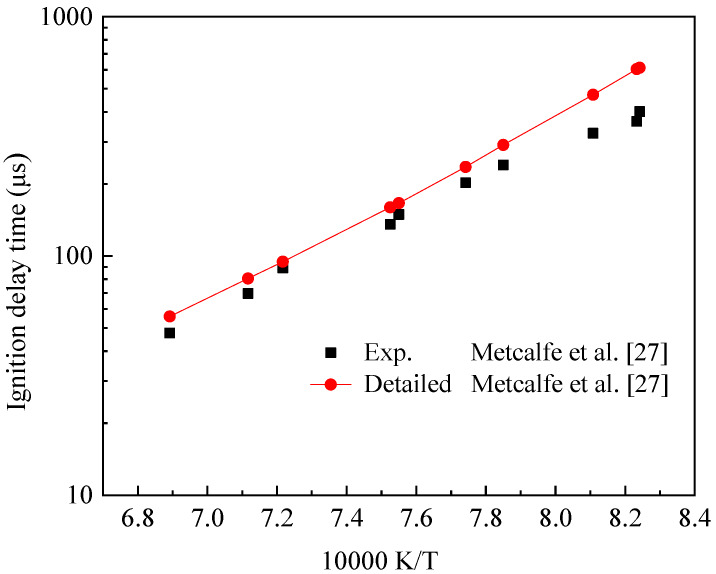
IC_8_H_16_ detailed mechanism verification.

The detailed chemical reaction kinetics of IC_8_H_16_ used in the simulation calculations was derived from the research results of Metcalfe et al. [27]. Figure 1 shows the verification of IC_8_H_16_ ignition delay time by Metcalfe et al. [27]. Under the conditions of equivalent ratio (*ϕ*) of 0.5 and pressure of 4.0 atm, the calculated ignition delay time of IC_8_H_16_ showed good agreement with the experimental value. The correctness of this detailed mechanism was verified.

#### 2.1.1. Analysis of the Reaction Path of IC_8_H_16_

To analyze the reaction path of IC_8_H_16_, we selected a single cylinder water-cooled direct injection engine as the numerical simulation object. The main technical parameters are shown in Table 1. The calculation start time was the intake valve closing (−141.5 °CA), and the calculation interval was 190 °CA. The mixture in the cylinder was assumed to be evenly distributed after the engine intake valve was closed. A homogeneous internal combustion engine model was selected in the zero-dimensional simulation software. An air/fuel equivalent ratio of 0.25 was selected to analyze the main reaction path of IC_8_H_16_, and the obtained main reaction path was added to the simplified mechanism of IC_8_H_16_ to be constructed.

**Figure 2:**
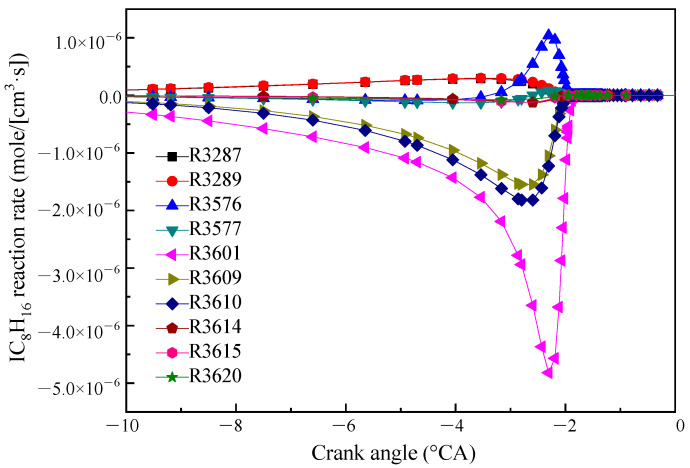
Reaction rate diagram of the main reaction of IC_8_H_16_.

The reaction rates of IC_8_H_16_ consumption are shown in Figure 2. IC_8_H_16_ was mainly consumed by the H-atom abstraction reactions by OH radical (R3609 and R3610) forming two isomers (IC_8_H_15_−A and JC_8_H_15_−B). In addition, the maximum reaction rate was the decarburization reaction (R3601). The products of R3601 are YC_7_H_13_−Y2 and CH_3_. The other reactions are ignored for the purpose of simplification. The main chemical reaction formulas are as follows:

R3601. IC_8_H_16_ = YC_7_H_13_ − Y2 + CH_3_ (1)

R3609. IC_8_H_16_ + OH = IC_8_H_15_ − A + H_2_O (2)

R3610. IC_8_H_16_ + OH = JC_8_H_15_ − B + H_2_O (3)

**Figure 3:**
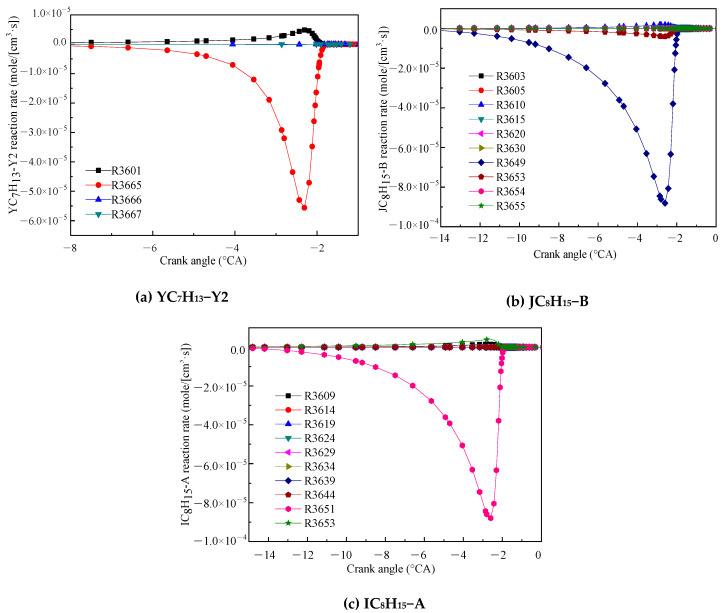
Reaction rates of the main reactions of the three products YC_7_H_13_−Y2 (**a**), JC_8_H_15_−B (**b**) and IC_8_H_15_−A (**c**).

Figure 3 shows the reaction rates for the related reactions of the three products of IC_8_H_16_. Figure 3a shows that the consumption reaction rates of YC_7_H_13_−Y2. YC_7_H_13_−Y2 is mainly consumed by the decomposition reaction (R3665). The products of R3665 are DMPD13 and H-atom. Figure 3b,c shows that the main consumption reaction of JC_8_H_15_−B and IC_8_H_15_−A were R3649 and R3651, respectively. The products of both reactions are DMPD13 and methyl. The other reactions are ignored for the purpose of simplification. The main chemical reaction formulas are as follows:

R3665. DMPD13 + H = YC_7_H_13_ − Y2 (4)

R3649. DMPD13 + CH_3_ = JC_8_H_15_ − B (5)

R3651. DMPD13 + CH_3_ = IC_8_H_15_ − A (6)

**Figure 4:**
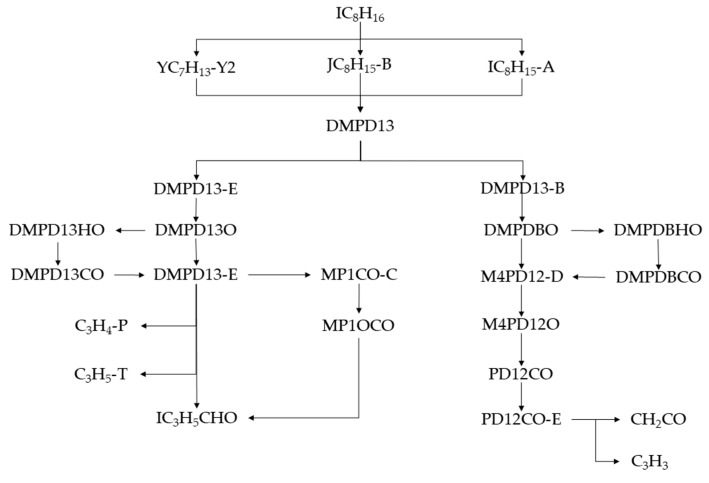
Reaction path of IC_8_H_16_.

IC_8_H_16_ undergoes dehydrogenation and decarburization reactions. Although different intermediate products exist, the same substance (DMPD13) was finally formed. In the next work, we continued to analyze the reaction path of DMPD13 and its products and finally obtained the reaction path of IC_8_H_16_, as shown in Figure 4.

#### 2.1.2. Temperature Sensitivity Analysis of IC_8_H_16_

In HCCI engine combustion, temperature is an important factor that affects fuel combustion. We used the temperature sensitivity analysis method to analyze the detailed mechanism of IC_8_H_16_. We also changed the initial temperature in the engine and shock tube models to find the response that had a greater effect on temperature changes.

**Figure 5:**
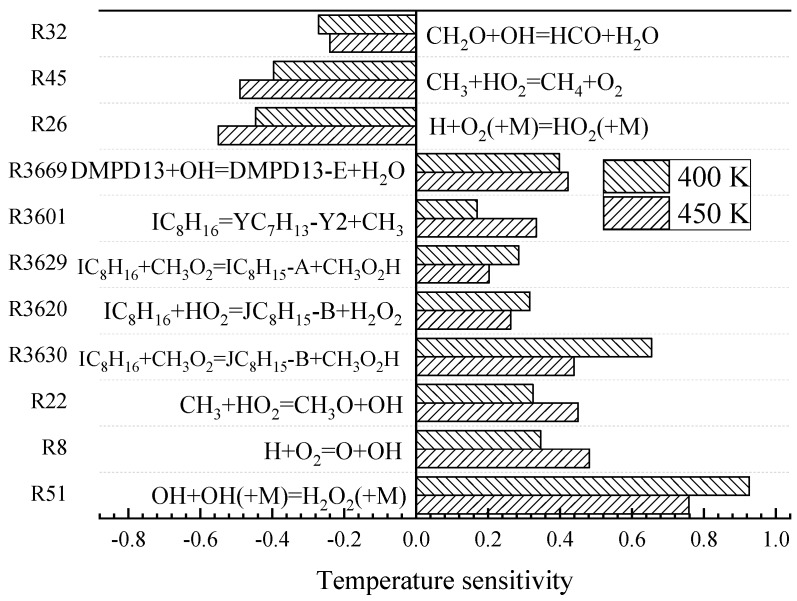
Temperature sensitivity coefficient of engine model simulation for IC_8_H_16_. Pressure 1.5 atm, *ϕ* = 0.25 and 400 and 450 K.

The engine parameters are shown in Table 1. The reactions with greater reaction temperature sensitivity were found at initial temperatures of 400 and 450 K. Figure 5 shows the result of temperature sensitivity analysis on IC_8_H_16_ under the conditions of HCCI engine with initial temperatures of 400 and 450 K. The pressure was 1.5 atm and the equivalent ratio *ϕ* was 0.25, the air used in the experiment was composed of 21% O_2_ and 79% N_2_. Eleven elementary reactions had a significant effect on the temperature in the engine cylinder (in Figure 5).

**Figure 6:**
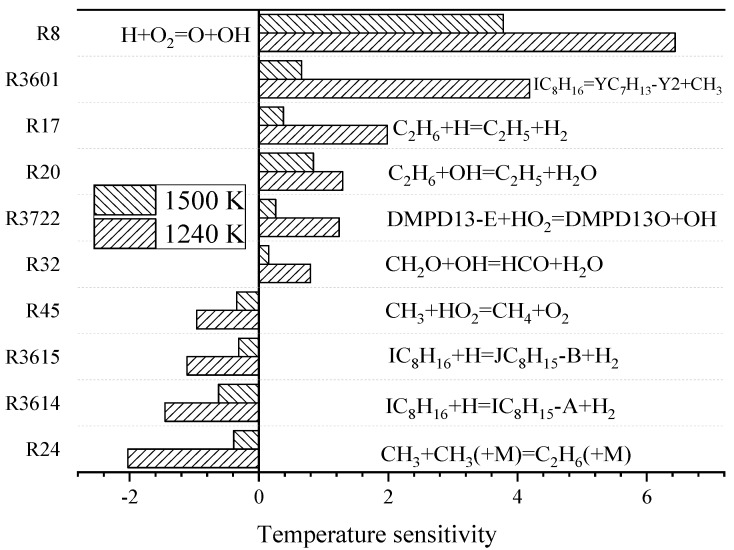
Temperature sensitivity coefficient simulated by shock tube model for IC_8_H_16_. Pressure 4.0 atm and 1240 and 1500 K.

Figure 6 shows the calculation results of temperature sensitivity in the shock tube model. The initial parameters of the model were as follows: the initial temperatures were 1240 and 1500 K, the pressure was 4 atm and the molar fractions of the mixed fuel components were IC_8_H_16_: 0.75% and O_2_: 18%, the rest of the tube was filled with inert gas Ar. In the temperature sensitivity analysis of the shock tube model, 10 elementary reactions with large sensitivity coefficients appeared and five of them (i.e., R17, R20, R3615, R3615 and R24) were new reaction.

The new reactions obtained from the temperature sensitivity analysis of the shock tube model were not all added to the simplified mechanism. Instead, different reaction combinations were applied. After adding reaction R3615, the simplified mechanism simulation results were compared and showed good agreement with the experimental results. The reaction mechanism of the formula was added as follows:

R3615. IC_8_H_16_ + H = JC_8_H_15_ − B + H_2_ (7)

### 2.2. NO Submechanism

**Figure 7:**
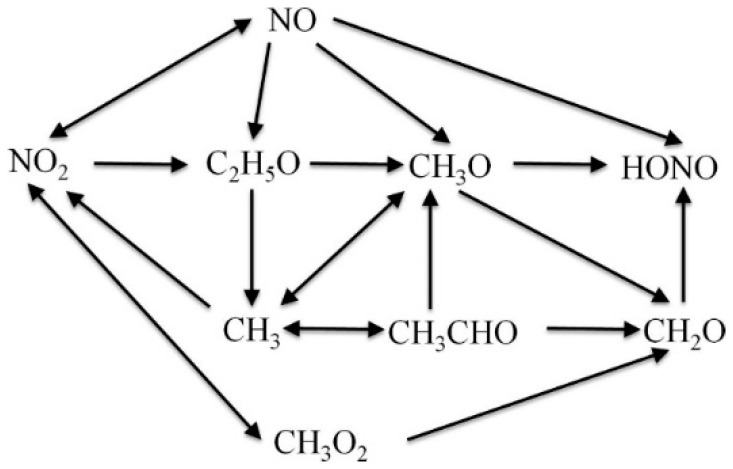
Reaction path of NO [24].

The effects of NO on the combustion process of *n*-heptane, isooctane and toluene have been analyzed by Zheng et al. [24], and a NO mechanism including 23 reactions has been constructed. Figure 7 shows the main reaction path for NO. The simulation showed that the active molecule NO changed some chemical reaction paths during fuel combustion and promoted the accumulation of OH at the initial stage of the reaction, thereby promoting the combustion.

The related reaction between NO and DIB was added on the basis of the NO submechanism proposed by Zheng et al. [24]. The reaction was combined with the TDRF simplified mechanism previously made into the TDRF–NO mechanism.

Contino et al. [23] found that NO and its derivatives have no effect on the reaction of DIB macromolecular fuel and mainly react with small molecules produced by the consumption of DIB. The mechanisms of the three substances C_3_H_3_, CH_4_ and CH_2_OH react with NO as follows:

C_3_H_3_ + N = HCN + C_2_H_2_ (8)

CH_3_ + HONO = CH_4_ + NO_2_ (9)

H_2_CNO_2_ + OH = CH_2_OH + NO_2_ (10)

Two new substances, namely, HCN and H_2_CNO_2_, appeared in the reaction mechanism. The reaction rate analysis of the two substances was required to find their main generation and reaction paths. The simulated HCCI engine parameters are shown in Table 1. The NO concentration was 50 ppmv.

**Figure 8:**
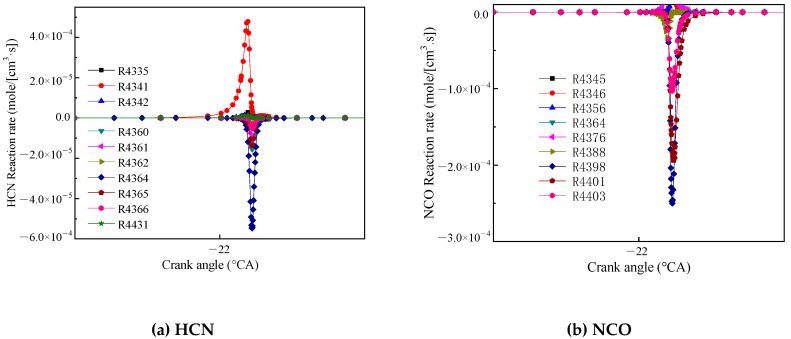
Main reaction rate of HCN (**a**) and NCO (**b**).

Figure 8a shows the reaction rate of HCN. The reaction R4341 (HCCO + NO = HCN + CO_2_) had the highest generation rate, and HCCO and NO reacted to form HCN. The reaction R4364 (HCN + O = NCO + H) had the highest consumption rate, and HCN oxidized NCO and generated a hydrogen (H). Figure 8b shows the main reaction rate of NCO. The reactions R4398 (NCO + O = NO + CO) and R4401 (NCO + M = N + CO + M) had the highest reaction rate, and NCO generated N, NO and CO.

**Figure 9:**
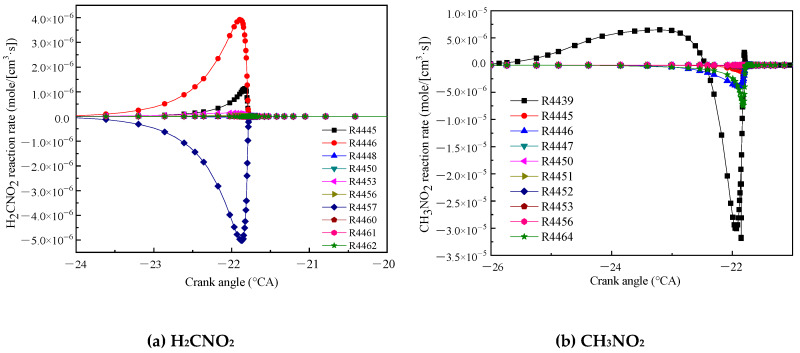
Main reaction rate of H_2_CNO_2_ (**a**) and CH_3_NO_2_ (**b**).

Figure 9a shows the reaction rate of H_2_CNO_2_. The reaction R4446 (CH_3_NO_2_ + OH = H_2_CNO_2_ + H_2_O) had the highest rate of formation, and CH_3_NO_2_ and OH reacted to dehydrogenate to form H_2_CNO_2_. The reaction R4457 (H_2_CNO_2_ = CH_2_O + NO) had the largest rate of consumption, and H_2_CNO_2_ was decomposed into CH_2_O and NO. Figure 9b shows the reaction rate of CH_3_NO_2_. The reaction R4439 (CH_3_NO_2_ [+M] = CH_3_ + NO_2_ [+M]) had the highest reaction rate. CH_3_ and NO_2_ combined to form CH_3_NO_2_. After the crank angle was −23 °CA, the reaction direction changed. CH_3_NO_2_ was decomposed into CH_3_ and NO_2_.

The related reactions obtained were combined with the NO mechanism proposed by Zheng et al. [24]; the mechanism constituted a NO mechanism including 33 reactions and was coupled with the simplified mechanism of TDRF produced earlier to include 266 reactions of 119 species. Finally, a simplified chemical kinetic mechanism of TDRF–NO was obtained (shown in Supplementary Material).

### 2.3. Validation of the Mechanism

Table 2 shows the volume ratio of each component of the surrogate fuels during the verification process.

The ability to predict the ignition delay time is an important indicator of the effectiveness of a chemical kinetic mechanism. Basing on previous studies, we constructed a simplified TDRF–NO chemical kinetic model containing five substances and verified the model and its submodels. All numerical simulations used the zero-dimensional single-region model as the calculation model in zero-dimensional software.

#### 2.3.1. TRF Submechanism Verification

**Figure 10:**
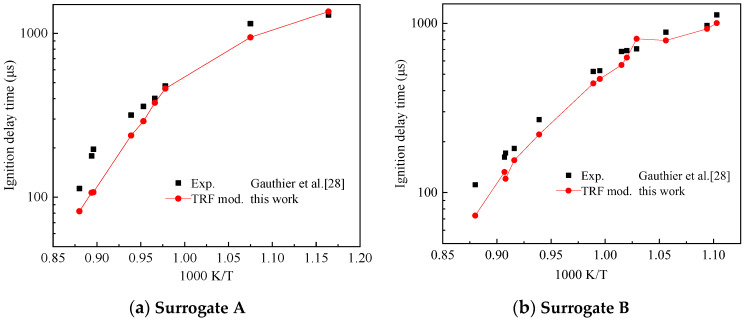
Comparison of experimental and toluene reference fuel (TRF) submechanism validation results on ignition delay times, (**a**) Surrogate A, (**b**) surrogate B.

The TRF mechanism used in this study was derived from the TRF part of the TDRF mechanism constructed by Zheng and Liang [25]. Gauthier et al. [28] performed shock tube experiments with various ratios of gasoline surrogates and obtained their ignition delay time. The gasoline surrogate TRF ratio used in the experiment is shown in Table 2 for Surrogate A and B. Figure 10 shows the comparison of the ignition delay simulation results of the simplified TRF mechanism with the experimental data of Gauthier et al. [28]. The initial temperature range of the ignition delay test was 850–1200 K. The pressure range of 1.5–6.0 MPa and the equivalent ratio *ϕ* was 1. The air used in the experiment was composed of 21% O_2_ and 79% N_2_.

Pressure scaling of all data points within a certain pressure range can unify the independent variables of the data displayed by the graph. The pressure scaling is generally assumed as a power function law (Equation (11)).

(11) $$\tau_{P^{*}}{=\tau (P}^{*}{/P)}^{-N}$$

where τp* is the ignition delay time from standard to pressure P*, *τ* is the ignition delay time, *P* is the experimental pressure, P* is the standard pressure and *N* is the pressure scaling factor.

Gauthier et al. [28] reported the following pressure scaling factors for two surrogates: mixed Surrogate A was 0.83 and Surrogate B was 0.96. Pressure scaling standard to 5.5 MPa. Figure 10 shows that the simplified mechanism can reflect the trend of the ignition delay time with the initial temperature for different ratios of TRFs well. This mechanism had a large error in the high-temperature conditions of the mixed Surrogate A and agreed well with the experimental results at low-temperature conditions (Figure 10a). The simulation results using the Surrogate B agreed well with the experimental results (Figure 10b). This mechanism can be used as the TRF part of the simplified mechanism of TDRF.

#### 2.3.2. DIB Submechanism Verification

**Figure 11:**
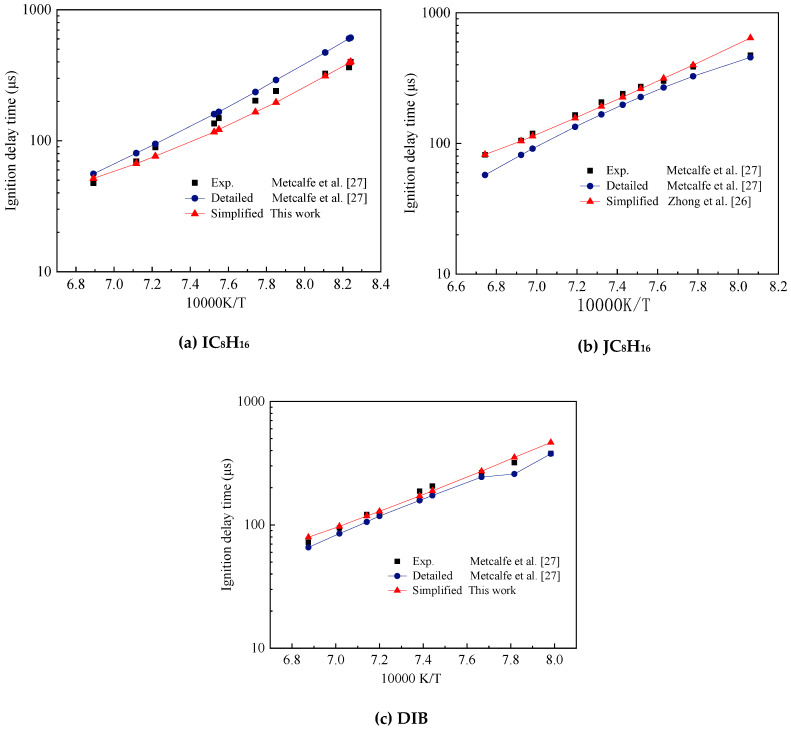
Comparison of experimental and diisobutylene (DIB) submechanism validation results on ignition delay times, (**a**) IC_8_H_16_, (**b**) JC_8_H_16_ and (**c**) DIB.

In this study, the mechanism of DIB was verified using shock tube experimental data obtained by Metcalfe et al. [27]. The simplified mechanism constructed was compared with the detailed mechanism to further illustrate the effectiveness of the mechanism. The comparison between the experimental data and the simplified and detailed mechanism simulation results of the shock tube in the temperature range of 1200–1500 K is shown in Figure 11. The experimental pressure was 4 atm and the equivalent ratio was 0.5. Table 3 shows the mole ratio of the two isomers of DIB. The simplified mechanism of JC_8_H_16_ used in the simulation was from Zhong et al. [26].

In Figure 11a, we compared the simplified and detailed mechanism of IC_8_H_16_ and experimental data of Fuel1. The general trend of the three sets of data were the same and the simplified mechanism and experimental data agreed well, especially around 1200 K to 1500 K.

Figure 11b shows the comparison of the simulation and experimental ignition delay of Fuel2. The simplified mechanism of JC_8_H_16_ used in the simulation is from Zhong et al. [26]. Most of the data of the simplified mechanism were consistent with the experimental results and the simulation error was smaller than the detailed mechanism.

Figure 11c is the comparison of the experimental and simulated ignition delay of Fuel3. The mole fraction ratio of JC_8_H_16_ and IC_8_H_16_ mixed fuel was 3:1. The simplification mechanism and experimental results agreed at different operating conditions, which indicated that the simulation results of the simplification mechanism of DIB constructed in this study were good and could be used to construct the TDRF simplification mechanism.

#### 2.3.3. TDRF-Simplified Mechanism Verification

In the previous work, we obtained a quaternary simplified mechanism containing 233 reactions of 133 species. The validity of the mechanism is verified below. This study used the experimental data of the ignition delay of the shock tube of quaternary gasoline substitute TDRF reported by Fikri et al. [17]. The fuel mixture (Surrogate D) used for the experiment ratio of the components is shown in Table 2.

**Figure 12:**
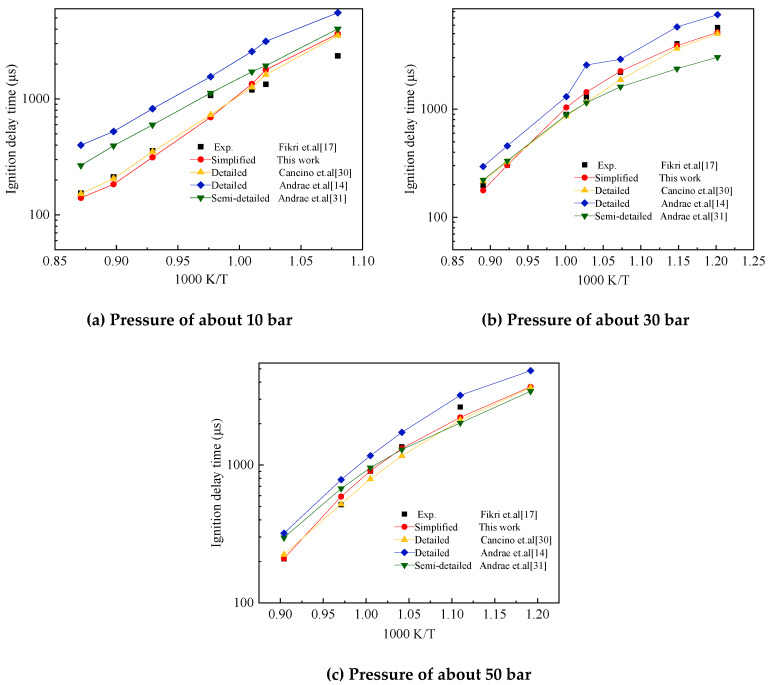
Comparison of experimental and the five-component gasoline surrogate fuel (TDRF) mechanism validation results on ignition delay times. Pressures of about (**a**) 10, (**b**) 30 and (**c**) 50 bar.

The initial temperature range of the shock tube experiment was 700–1200 K. The experimental pressure was about 10, 30 and 50 bar and the equivalent ratio *ϕ* was 1. The air used in the experiment was composed of 21% O_2_ and 79% N_2_. Figure 12 shows the comparison of the ignition delay times between the experimental results in shock tube by Fikri et al. [17] and the four computational results obtained using the detailed mechanism of Cancino et al. [30], the detailed and semidetailed mechanisms of Andrea et al. [14,31], and the simplified mechanism of TDRF proposed in the present study. The pressure standard was set to 30 bars. The pressure scaling factor was 0.65.

Figure 12 also shows that the four mechanisms can reflect the trend of ignition delay time with temperature well around three initial pressures. The simplified mechanism of TDRF constructed in the present study and the detailed mechanism of Cancino et al. [30] showed a good agreement with the experimentally measured ignition delay time as a whole. The simplified model can show the change of ignition delay time with the initial temperature and pressure of the shock tube well.

#### 2.3.4. TDRF–NO-Simplified Mechanism Verification

Finally, the TDRF mechanism coupled with the NO submechanism constituted a simplified TDRF–NO chemical kinetic mechanism with 119 species and 266 reactions (shown in Supplementary Material).

Pera et al. [29] studied the ratio of ternary TRF fuel to gasoline and obtained the proportion of each component of the TRF fuel (Surrogate C), as shown in Table 2. This ratio was obtained from the H/C ratio of the blended gasoline and the octane number. The proportion of each component of TDRF fuel (Surrogate D) in Table 2 was from Fikri et al. [17].

**Figure 13:**
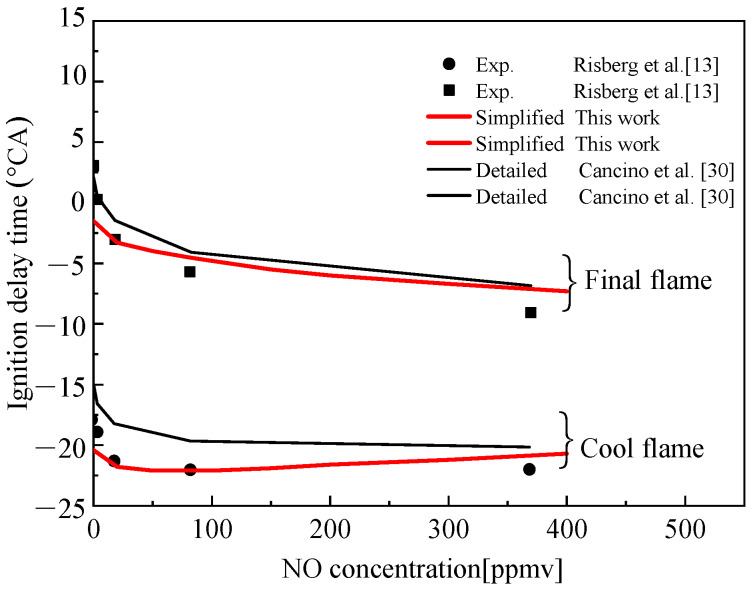
Comparison of simulated and experimental values of the ignition delay time of Surrogate C with the concentration of NO added.

Figure 13 presents the comparison of the calculated values of the simplified mechanism of TDRF–NO constructed by using Surrogate C and the detailed mechanism of Cancino et al. [30] with the experimental values of gasoline experiments under the experimental conditions of Risberg et al. [13], and it also shows that both models could simulate the effect of NO on gasoline fuel combustion at this ratio. However, the data of the simplified mechanism were consistent with the experimental results and the simulation error was smaller than the detailed mechanism.

**Figure 14:**
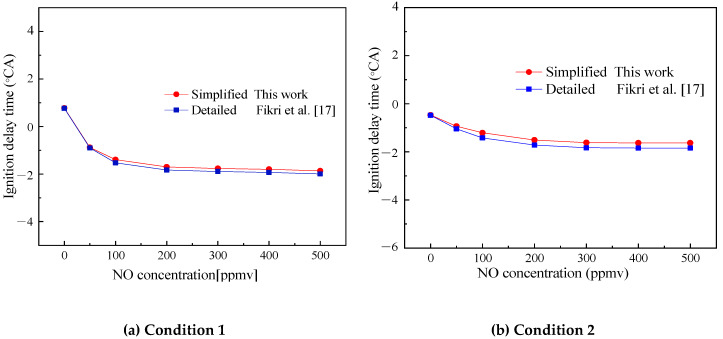
Comparison of simulated and experimental values of the ignition delay time of Surrogate D with the concentration of NO added. (**a**) Condition 1, *ϕ* = 0.25, 4 atm and 600 K; (**b**) Condition 2, *ϕ* = 0.25, 2 atm and 800 K.

Figure 14 shows the comparison of the ignition delay time of Surrogate D under TDRF-simplified and detailed mechanisms with the concentration of NO added under two design conditions. The two conditions are as follows: Condition 1, *ϕ* = 0.25, initial pressure of 4 atm and initial temperature of 600 K; Condition 2, *ϕ* = 0.25, initial pressure of 2 atm and initial temperature of 800 K.

Given that no experimental data of TDRF–NO are available, this article only compared the simplified and detailed mechanisms. Figure 14 shows that the simplified and detailed mechanism simulations had the same trend with increasing NO concentration. As the concentration of NO increased, the ignition increased. The simplified and detailed mechanisms agreed well at each operating point, whereas the simplified mechanism had only 134 reactions. Compared with the detailed mechanism, the simplified mechanism is within the error range required by simulation and it could simulate the combustion of fuel and saved much of the calculation costs, reflecting the superiority of the simplified mechanism.

## 3. Materials and Methods

### 3.1. Zero-Dimensional Simulation Software

**Figure 15:**
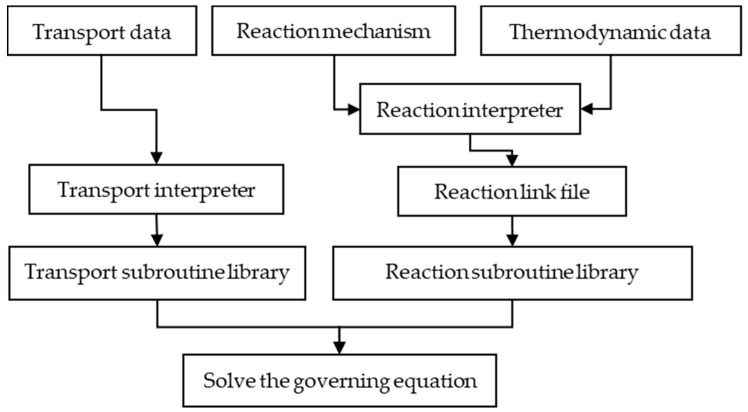
The solution flow chart of zero-dimensional simulation software.

This study used zero-dimensional simulation software (Sandia National Laboratories, Albuquerque, NM, USA, 1980) to calculate the fuel combustion process. Figure 15 shows the flow of the simulation solution process. The thermodynamic data were read by the preprocessor of the gas phase dynamics to generate a link file that containing elements, components and their thermodynamic data information. The reaction mechanism was read by the surface dynamics preprocessor (in software) to form a link file containing surface reaction information. The preprocessor of the transfer process automatically reads the transfer data from the database in software based on the information in the gas dynamics connection file to generate a transfer connection file containing the transfer information.

The transport subroutine library (in software) and the reaction subroutine library (in software) store the above-mentioned transport data and the information in the link file into a matrix, and then calculated the corresponding physical parameters. Finally, the governing equations were solved.

### 3.2. Source of Kinetic Mechanisms

The TRF mechanism used in this study was derived from the TRF part of the TDRF mechanism constructed by Zheng and Liang [25]. The gasoline surrogate TRF ratio used in the experiment is Surrogate A (17% *n*-heptane/63% isooctane/20% toluene by liquid volume), Surrogate B (17% *n*-heptane/69% isooctane/14% toluene by liquid volume) and Surrogate C (17% *n*-heptane/69% isooctane/14% toluene by liquid volume).

DIB is a mixture of two conjugated olefins, JC_8_H_16_ and IC_8_H_16_. The detailed chemical reaction kinetics of IC_8_H_16_ used in the simulation calculations was derived from the research results of Metcalfe et al. [27] who used a mechanism that includes 897 species and 3783 reactions. The simplified mechanism of JC_8_H_16_ used in the simulation is from Zhong et al. [26].

The gasoline surrogate TDRF ratio used in the experiment is Surrogate D (25% *n*-heptane/20% isooctane/45% toluene/10% diisobutene by liquid volume).

The NO submechanism was based on the report of Zheng et al. [24] NO submechanism. The related reaction between NO and DIB was added on the basis of the NO submechanism proposed by Zheng et al. The reaction was combined with the TDRF simplified mechanism into the TDRF–NO mechanism.

### 3.3. Simplified Calculation Method

#### 3.3.1. Rate of Production Analysis

Rate of Production (ROP) analysis was to analyze the effect of the elementary reaction on the net generation rate of a component, so that several important elementary reactions on the component can be obtained relatively quickly. The calculation formula of the chemical reaction generation rate $P_{k}$ and the influence coefficient ${\bar{C}}_{ki}^{p}$ of the chemical reaction generation rate was as follows:

(12) $$P_{k}={\overset{\cdot }{\omega }}_{k}=\overset{I}{\underset{i=1}{\sum }}\nu_{ki}q_{i}$$

(13) $${\bar{C}}_{ki}^{p}=\frac{{max(\nu }_{\mathrm{ki}},0)}{\sum_{i=1}^{I}{max(\nu }_{ki}{,0)q}_{i}}$$

where $q_{i}$ represents the chemical reaction rate of the i-th element reaction,$\;\nu_{ki}$ represents the chemical equivalent coefficient, $\nu_{ki}q_{i}$ represents the effect of the chemical reaction rate component *k* generation rate of the i-th element reaction and *I* represents all the number of elementary reactions containing component *k*.

During the reaction, the ROP coefficient ${\bar{C}}_{ki}^{p}$ can be used to compare the effect of the elementary reaction on the net formation rate of the component. When ${\bar{C}}_{ki}^{p}$ was positive, it means that the elementary reaction promotes the formation of a certain component. When ${\bar{C}}_{ki}^{p}$ was negative, it means that the elementary reaction promotes the consumption of a certain component.

#### 3.3.2. Sensitivity Analysis

The sensitivity analysis method was used to analyze and obtain the reaction with greater sensitivity to the parameter changes in the reaction system and to know whether a certain elementary reaction promotes the fuel combustion process. The sensitivity orthogonal coefficient ${\tilde{S}}_{L}$ of the j-th elementary reaction can be obtained by the following formula:

(14) $${\tilde{S}}_{L}=\frac{k_{j}}{c_{i}}\frac{\partial c_{i}}{\partial k_{j}}=\frac{\partial lnc_{i}}{\partial lnk_{j}}$$

where $c_{i}$ represents the chemical reaction rate constant of the j-th element reaction in the chemical reaction mechanism and $k_{j}$ represents the concentration of the i-th substance in the chemical reaction mechanism.

### 3.4. Mechanism Verification Method

The ability to predict the ignition delay time is an important indicator of the effectiveness of a chemical kinetic mechanism. In this study, the mechanism was verified using shock tube experimental data obtained by previous research.

## 4. Conclusions

A component DIB was added based on the simplified mechanism of TRF to construct a simplified mechanism of TDRF. Considering the effect of active molecular NO on the combustion process of gasoline fuel, we constructed a NO submechanism. A simplified TDRF–NO chemical kinetic mechanism consisting of 119 species and 266 reactions was formed by coupling the TDRF mechanism with the NO submechanism. The simplified mechanism obtained satisfactory results in the verification of the ignition delay in the shock tube.

(1) A complete simplified IC_8_H_16_ mechanism was obtained by analyzing the reaction path and temperature sensitivity of IC_8_H_16_. The simplified mechanism of IC_8_H_16_ was coupled with the simplified mechanism of JC_8_H_16_ to construct a DIB submechanism. The new mechanism was compared with the experimental data and detailed mechanism, and comparison results show that the consistency of the three models was better.
(2) The NO submechanism was based on our previous research of NO submechanism and related reactions of NO and DIB were added to construct a NO submechanism containing 33 reactions.
(3) The TRF mechanism was derived from the TRF part of the TDRF mechanism that we constructed before and the simplified mechanism of TDRF was formed by coupling the simplified mechanism of DIB. The ignition delay time was verified under the condition of a shock tube. The model verification results were in good agreement with the experimental data.
(4) The TDRF and NO submechanisms were coupled into a TDRF–NO chemical kinetic simplified mechanism. The two fuels were compared with the detailed mechanisms and experimental data. The ignition delay time of the simplified and detailed mechanism simulations increased with increasing NO concentration. The reliability of TDRF–NO simplified mechanism was illustrated.

## Figures and Tables

**Table 1:** Main engine parameters.

Index Item	Value
Compression ratio	17.5
Bore (mm)	112
Stroke (mm)	132
Displacement (cm^3^)	1300
Speed (rpm)	1400
Crankshaft radius ratio	3.714
Temperature (K)	400
Pressure (atm)	1.5
Intake valve closing time (°CA)	38.5

**Table 2:** Volume ratios of various components of several gasoline surrogate fuels.

Fuel	Isooctane	Toluene	*n*-heptane	Diisobutylene	Ref.
Surrogate A	63%	20%	17%	-	[28]
Surrogate B	69%	14%	17%	-	[28]
Surrogate C	13.7%	34.8%	51.5%	-	[29]
Surrogate D	20%	45%	25%	10%	[17]

**Table 3:** Mole fractions of 2,4,4-trimethyl-1-pentene and 2,4,4-trimethyl-2-pentene (balance is argon) [27].

Fuel	JC_8_H_18_	IC_8_H_16_	O_2_	Ar
Fuel1	-	0.75%	18%	81.25%
Fuel2	0.75%	-	18%	81.25%
Fuel3	0.56%	0.19%	18%	81.25%

## Supplementary Materials

The following are available online. Table S1: TDRF–NO simplified mechanism.
