# Supplementary material for: Chemical Kinetic Model of Multicomponent Gasoline Surrogate Fuel with Nitric Oxide in HCCI Combustion
Source: Molecules. 2020 May 12;25(10):2273. doi: 10.3390/molecules25102273 (PMC7287883; doi:10.3390/molecules25102273)
Supplement: Supplementary file 1 [file molecules-25-02273-s001.pdf]

Table S1：TDRF-NO simplified mechanism

Species				
$AC_8H_{16}OOH$	C_4H_5	CH_3	H_2CNO_2	MPT124
AC_8H_{17}	C_5H_{11}	CH_3CHO	H_2O	MPT124J
$AC_8H_{17}O$	C_5H_4O	CH_3CO	H_2O_2	N
$AC_8H_{17}O_2$	$C_6H_4CH_3$	CH_3COCH_2	HCCO	N_2
$AOOC_8H_{16}OOH$	C_6H_4O2	CH_3NO_2	HCN	NC_7H_{16}
AR	C_6H_5	CH_3O	HCO	NCO
MP1OCO	$C_6H_5CH_2$	CH_3O_2	HO_2	NO
MP1CO-C	$C_6H_5CH_2O$	CH_3O_2H	H	NO_2
$C_6H_5CH_2OO$	C_2H	CH_3OH	$HOC_6H_4CH_3$	O
$HO_2C_7H_{13}O_2H$	$C_6H_5CH_3$	CH_4	HONO	O_2
C_2H_3	C_6H_5CHO	CO	IC_3H_5CHO	$OC_6H_4CH_3$
C_2H_4	C_6H_5CO	CO_2	IC_3H_5CO	$OC_7H_{13}O$
C_2H_5	C_6H_5O	DMBD13-D	IC_3H_7	$OC_7H_{13}OOH$
C_2H_5O	C_6H_5OH	DMPD13	IC_4H_7	$OC_8H_{15}O$
$C_2H_5O_2$	$C_7H_{14}OOH$	DMPD13-B	IC_4H_7O	$OC_8H_{15}OOH$
C_2H_6	C_7H_{15}-1	DMPD13CO	IC_4H_8	OH
C_3H_3	C_7H_{15}-2	DMPD13-E	IC_8H_{15}-A	$OOC_7H_{14}OOH$
C_3H_4	$C_7H_{15}O$	DMPD13HO	IC_8H_{16}	PC_4H_9
C_3H_4-A	$C_7H_{15}OO$	DMPD13O	IC_8H_{18}	PD12CO
C_3H_4-P	CH	DMPD13OHE	JC_8H_{15}-B	PD12CO-E
C_3H_5	CH_2	DMPDBCO	JC_8H_{16}	TC_4H_9
C_3H_5-T	CH_2CHO	DMPDBHO	M4PD12-D	YC_7H_{13}-Y2
C_3H_6	CH_2CO	DMPDBO	M4PD12O	C_4H_4
C_4H_3	CH_2O	H_2	CH_2OH	

No.	Reactions	A	b	Ea
$k = AT^b \exp(-E_a/RT)$				
2	NC_7H_{16}+ O_2=> C_7H_{15}-1+ HO_2	6.00E+13	0	52820
3	NC_7H_{16}+ O_2=> C_7H_{15}-2+ HO_2	4.00E+13	0	50190
4	NC_7H_{16}+ HO_2=> C_7H_{15}-1+ H_2O_2	5.00E+13	0	20430
5	NC_7H_{16}+ HO_2=> C_7H_{15}-2+ H_2O_2	3.36E+13	0	17690
6	NC_7H_{16}+OH=> C_7H_{15}-1+ H_2O	1.05E+10	1	1590
7	NC_7H_{16}+OH=> C_7H_{15}-2+ H_2O	9.40E+07	1.6	-35
8	C_7H_{15}-1=> C_7H_{15}-2	2.00E+11	0	18120
9	C_7H_{15}-2=> C_7H_{15}-1	2.00E+11	0	18120
10	C_7H_{15}-1+ O_2=> $C_7H_{15}OO$	2.50E+12	0	0
11	$C_7H_{15}OO$=> C_7H_{15}-1+ O_2	2.20E+15	0	27960

12	C_7H_{15}-2+ O_2=> $C_7H_{15}OO$	2.50E+12	0	0
13	$C_7H_{15}OO$=> C_7H_{15}-2+ O_2	2.20E+15	0	26960
14	$C_7H_{15}OO$=> $C_7H_{14}OOH$	2.00E+11	0	17010
15	$C_7H_{14}OOH$ + O_2=>OO $C_7H_{14}OOH$	5.60E+12	0	0
16	OO $C_7H_{14}OOH$ => $HO_2C_7H_{13}O_2H$	2.00E+11	0	17010
17	$HO_2C_7H_{13}O_2H$=> $OC_7H_{13}OOH$+OH	1.00E+09	0	7500
18	$OC_7H_{13}OOH$=> $OC_7H_{13}O$ +OH	8.40E+14	0	43020
19	$OC_7H_{13}O$ =>C H_2O+ C_5H_{11}+CO	2.00E+13	0	15000
20	C_7H_{15}-1=> C_5H_{11}+ C_2H_4	2.50E+13	0	28920
21	C_7H_{15}-2=> PC_4H_9+ C_3H_6	1.20E+13	0	28203
22	C_5H_{11}=> C_2H_4+ IC_3H_7	7.97E+17	-1.4	29876
23	NC_7H_{16}+H=> C_7H_{15}-1+ H_2	1.88E+05	2.8	6286
24	NC_7H_{16}+H=> C_7H_{15}-2+ H_2	2.60E+06	2.4	4469
25	IC_3H_7+ O_2= C_3H_6+ HO_2	6.10E+20	-2.9	7910
26	C_3H_6+H(+M)= IC_3H_7 (+M)	5.70E+09	1.2	874
	Low pressure limit: 1.64E+54 -1.11E+01 9.36E+03			
	TROE centering: 1.00E+00 1.00E-15 2.60E+02 3.00E+03			
H_2O	Enhanced by 5.00E+00			
H_2	Enhanced by 2.00E+00			
CO2	Enhanced by 3.00E+00			
CO	Enhanced by 2.00E+00			
27	IC_3H_7+H= C_2H_5+ CH_3	5.00E+13	0	0
28	PC_4H_9 (+M)= C_2H_5+ C_2H_4 (+M)	1.06E+13	0	27828
	Low pressure limit: 1.90E+55 -1.19E+01 3.23E+04			
H_2O	Enhanced by		5.00E+00	
H_2	Enhanced by		2.00E+00	
CO2	Enhanced by		3.00E+00	
CO	Enhanced by		2.00E+00	
29	PC_4H_9= C_2H_5+ C_2H_4	2.50E+13	0	28800
30	IC_8H_{18}+O_2= AC_8H_{17}+ HO_2	1.00E+16	0	49000
Reverse Arrhenius coefficients:		1.00E+12	0	0
31	IC_8H_{18}+ HO_2= AC_8H_{17}+ H_2O_2	3.02E+12	0	14700
32	AC_8H_{17}+O_2= $AC_8H_{17}O_2$	1.00E+12	0	0
Reverse Arrhenius coefficients:		2.51E+13	0	27400
33	$AC_8H_{17}O_2$= $AC_8H_{16}OOH$	1.14E+11	0	22400
Reverse Arrhenius coefficients:		1.00E+11	0	11000
34	AC8H16OOH+O_2= $AOOC_8H_{16}OOH$	3.16E+11	0	0
Reverse Arrhenius coefficients:		2.51E+13	0	27400
35	$AOOC_8H_{16}OOH$ => $OC_8H_{15}OOH$+OH	8.91E+10	0	17000
36	IC_8H_{18}+OH=> AC_8H_{17}+ H_2O	3.00E+13	0	3000
37	$OC_8H_{15}OOH$=> $OC_8H_{15}O$ +OH	3.98E+15	0	43000
38	AC_8H_{17}+O_2= JC_8H_{16}+ HO_2	3.16E+11	0	6300
Reverse Arrhenius coefficients:		3.16E+11	0	19500

39	$OC_8H_{15}O+O_2$=> $C_2H_3+2CH_2O+C_3H_4+CH_3+HO_2$	2.45E+13	0	32000
40	AC_8H_{17}=> $IC_4H_8+ C_3H_6+ CH_3$	1.28E+12	0	49000
41	JC_8H_{16}=> $IC_4H_8+ C_3H_5+ CH_3$	1.92E+12	0	49000
42	$IC_4H_8+ O_2$=> $C_2H_3+ C_2H_4+ HO_2$	2.00E+14	0	35900
43	$IC_4H_8= C_3H_5+ CH_3$	1.92E+66	-14.2	128100
Reverse Arrhenius coefficients:		2.09E+59	-13.2	29530
44	$C_6H_5CH_3+ O_2= C_6H_5CH_2+ HO_2$	1.81E+12	0	39717
45	$C_6H_5CH_3+OH= C_6H_5CH_2+ H_2O$	6.70E+09	1	870
46	$C_6H_5CH_3+O= C_6H_5CH_2+OH$	6.30E+11	0	0
47	$C_6H_5CH_3+H= C_6H_5CH_2+ H_2$	6.00E+13	0	8235
48	$C_6H_5CH_2+O= C_6H_5CHO+H$	2.50E+14	0	0
49	$C_6H_5CH_2+O= C_6H_5+CH_2O$	8.00E+13	0	0
50	$C_6H_5CH_2+ HO_2= C_6H_5CHO+H+OH$	2.60E+13	0	0
51	$C_6H_5CH_2+ HO_2= C_6H_5CHO+ H_2O$	7.50E+12	0	0
52	$C_6H_5CH_2+ HO_2= C_6H_5+CH_2O+OH$	6.00E+12	0	0
53	$C_6H_5CH_2+H= C_6H_5CH_3$	1.80E+14	0	0
54	$C_6H_5CHO+OH= C_6H_5CO+ H_2O$	1.71E+09	1.2	-447
55	$C_6H_5CHO+H= C_6H_5CO+ H_2$	5.00E+13	0	4928
56	$C_6H_5CHO+O= C_6H_5CO+OH$	9.04E+12	0	3080
57	$C_6H_5CH_2+ C_6H_5CHO= C_6H_5CH_3+ C_6H_5CO$	2.77E+03	2.8	5773
58	$C_6H_5CO= C_6H_5+CO$	3.98E+14	0	29400
59	$C_6H_5+O_2=C\ H_2CO+CH_2CO+C_2H$	7.80E+16	-1.8	0
60	$C_6H_5+O_2= C_6H_5O+O$	2.60E+13	0	6100
61	$C_6H_5+OH= C_6H_5O+H$	5.00E+13	0	0
62	$C_6H_5OH+OH= C_6H_5O+ H_2O$	3.00E+06	2	-1310
63	$C_6H_5CH_2+ C_6H_5OH= C_6H_5CH_3+ C_6H_5O$	1.05E+12	0	9500
64	$C_6H_5+O_2= C_6H_4O_2+H$	3.00E+13	0	8980
65	$C_6H_5O+O= C_6H_4O_2+H$	5.00E+13	0	0
66	$C_6H_5O+ HO_2= C_6H_4O_2+ H_2O$	1.00E+13	0	0
67	$C_6H_4O_2+O$=>$2CO+C_2H_2+CH_2CO$	3.00E+13	0	5000
68	$C_6H_5CH_3+OH= C_6H_4CH_3+H_2O$	3.40E+08	1.4	1450
69	$C_6H_4CH_3+O_2=O\ C_6H_4CH_3+O$	2.60E+13	0	6100
70	$HO\ C_6H_4CH_3+H= C_6H_5CH_3+OH$	2.21E+13	0	7910
71	$O\ C_6H_4CH_3+H=HOC_6H_4CH_3$	2.50E+14	0	0
72	$HO\ C_6H_4CH_3+OH=OC_6H_4CH_3+ H_2O$	6.00E+12	0	0
73	C_5H_4O +O= C_4H_4+CO2	1.00E+13	0	2000
74	C_4H_5+O2=> $C_4H_4+ HO_2$	1.00E+08	2	10000
75	$C_4H_4+OH= C_4H_3+ H_2O$	7.50E+06	2	5000
76	$C_4H_4+O=C_3H_3+HCO$	8.10E+06	1.9	180
77	$C_4H_3+O_2=C_2H_2+CO+HCO$	3.00E+12	0	0
78	C_7H_{15}-1+ $C_6H_5CH_3= C_6H_5CH_2+ NC_7H_{16}$	1.00E+11	0	12000
79	C_7H_{15}-2+ $C_6H_5CH_3= C_6H_5CH_2+ NC_7H_{16}$	1.00E+11	0	12000
80	$AC_8H_{17}+ C_6H_5CH_3= C_6H_5CH_2 + IC_8H_{18}$	1.00E+11	0	12000

81	IC_8H_{18}+ C_7H_{15}-1= NC_7H_{16}+ AC_8H_{17}	9.00E+11	0	13500
82	IC_8H_{18}+ C_7H_{15}-2= NC_7H_{16}+ AC_8H_{17}	9.00E+11	0	14500
83	C_3H_6+H= C_2H_4+ CH_3	7.20E+12	0	1300
84	C_3H_6+O= C_3H_5+OH	5.20E+11	0.7	5884
85	C_3H_6+OH= C_3H_5+ H_2O	3.10E+06	2	1451
86	C_3H_6+OH=> C_2H_5+C H_2O	7.90E+12	0	0
87	C_3H_6+O_2= C_3H_5+ HO_2	2.00E+13	0	47600
88	C_3H_5+O_2=CH_2CHO+CH_2O	1.10E+10	0.3	12840
89	C_3H_5+O_2= C_3H_4+HO_2	5.00E+15	-1.4	22430
90	C_3H_5+OH= C_3H_4+ H_2O	2.00E+13	0	0
91	C_3H_4+OH= C_2H_3+C H_2O	1.00E+12	0	0
92	C_3H_4+OH= C_2H_4+HCO	1.00E+12	0	0
93	C_2H_5+O=CH3CHO+H	5.30E+13	0	0
94	C_2H_5+OH= C_2H_4+ H_2O	2.40E+13	0	0
95	C_2H_5+O_2=> C_2H_4+ HO_2	1.00E+10	0	-2190
96	C_2H_4+H= C_2H_3+ H_2	5.40E+14	0	14900
97	C_2H_4+H(+M)= C_2H_5 (+M)	1.10E+12	0.5	1822
Low pressure limit: 1.11E+34 -5.00E+00 4.45E+03				
TROE centering: 5.00E-01 9.50E+01 9.50E+01 2.00E+02				
H_2O	Enhanced	by	5.00E+0	
98	C_2H_4+OH=> C_2H_3+ H_2O	8.02E+13	0	5955
99	C_2H_3+O_2=C H_2O+HCO	1.10E+23	-3.3	3890
100	C_2H_3+O_2=C H_2CHO+O	2.50E+15	-0.8	3135
101	C_2H_2+O_2=>HCCO+OH	2.00E+08	1.5	30100
102	C_2H_2+OH=> CH_3+CO	2.00E+07	2	3214.3
103	C_2H_2+O=HCCO+H	1.40E+07	2	1900
104	C_2H_2+O=CH_2+CO	6.10E+06	2	1900
105	C_2H+O_2=CO+CO+H	2.50E+13	0	0
106	HCCO+O_2=>HCO+CO+O	9.78E+11	0	850
107	HCCO+O_2=>HCO+CO_2	6.52E+11	0	850
108	HCCO+O_2=CO+CO+OH	2.90E+07	1.7	1000
109	CH_3CHO+OH= CH_3CO+ H_2O	2.30E+10	0.7	-1110
110	CH_3CO(+M)= CH_3+CO(+M)	2.80E+13	0	17100
Low pressure limit: 2.10E+15 0.00E+00 1.40E+04				
TROE centering: 5.00E-01 1.00E-30 1.00E+30				
H_2	Enhanced	by		2.00E+00
CO	Enhanced	by		2.00E+00
CO_2	Enhanced	by		3.00E+00
H_2O	Enhanced	by		5.00E+00
111	CH_2CHO+O_2=CH_2O+CO+OH	2.20E+11	0	1500
112	CH_2CO+OH=HCCO+ H_2O	1.00E+07	2	3000
113	CH_3+O=C H_2O+H	8.40E+13	0	0
114	CH_3+O_2=C H_2O+OH	1.90E+12	0	20315
115	CH_3+ HO_2= CH_3O+OH	8.00E+12	0	0

116	CH_3+H(+M)=CH4(+M)	1.30E+16	-0.6	383
Low pressure limit:	1.75E+33 -4.76E+00 2.44E+03			
TROE centering:	7.83E-01 7.40E+01 2.94E+03 6.96E+03			
H_2O	Enhanced	by		8.57E+00
N_2	Enhanced	by		1.43E+00
117	CH_3+CH_2O=CH4+HCO	7.80E-08	6.1	1967
118	CH_2O+H(+M)= CH_3O(+M)	5.40E+11	0.5	2600
Low pressure limit:	1.54E+30 -4.80E+00 5.56E+03			
TROE centering:	7.58E-01 9.40E+01 1.56E+03 4.20E+03			
N_2	Enhanced	by		1.43E+00
H_2O	Enhanced	by		8.58E+00
119	CH_2O+OH=HCO+ H_2O	3.40E+09	1.2	-447
120	CH_2O+O_2+M=>H+CO+M+ HO_2	6.20E+16	0	36939.9
121	CH_2O+ HO_2=>HCO+ H_2O_2	2.17E+11	0	7987
122	CH_2+OH=CH+ H_2O	1.13E+07	2	3000
123	CH_2+O_2=CO+ H_2O	2.20E+22	-3.3	2867
124	CH_2+H=CH+ H_2	1.00E+18	-1.6	0
125	CH_2+O_2=CO+OH+H	1.60E+21	-3.3	2867
126	CH_2+ H_2=H+ CH_3	5.00E+05	2	7230
127	CH_2+CO_2=C H_2O+CO	1.10E+12	0	1000
128	HCO+O_2= HO_2+CO	7.60E+12	0	406
129	HCO+M=H+CO+M	1.90E+17	-1	17000
H_2O	Enhanced	by		5.00E+00
130	HCO+H=CO+ H_2	1.20E+13	0.2	0
131	HCO+OH= H_2O+CO	1.00E+14	0	0
132	CO+ HO_2=>CO_2+OH	1.51E+14	0	23611.6
133	CO+O=CO_2	1.80E+10	0	2434
134	CO+O+M=CO_2+M	5.89E+15	0	4093.3
135	CO+OH=CO_2+H	3.09E+11	0	733.8
136	CO_2+O=CO+O_2	2.75E+12	0	43758.8
Reverse Arrhenius coefficients:		3.25E+11	0	36610.4
137	CH_3OH+OH=CH_3O+ H_2O	1.00E+06	2.1	496.7
Reverse Arrhenius coefficients:		8.98E+06	2.1	17380
138	O+OH=O_2+H	2.00E+14	-0.4	0
139	O+ H_2=OH+H	5.00E+04	2.7	6290
140	OH+ H_2= H_2O+H	2.10E+08	1.5	3450
141	2OH=O+ H_2O	4.30E+03	2.7	-2486
142	H+H+M= H_2+M	1.00E+18	-1	0
H_2O	Enhanced	by		0.00E+00
143	H+H+ H_2O= H_2+ H_2O	6.00E+19	-1.2	0
144	H+O+M=OH+M	6.20E+16	-0.6	0
H_2O	Enhanced	by		5.00E+00
145	H+OH+M= H_2O+M	1.60E+21	-2	0
H_2O	Enhanced	by		5.00E+00

146	O+O+M=O2+M	1.90E+13	0	-1788
H_2O	Enhanced	by		5.00E+00
147	$H+O_2+M= HO_2+M$	2.10E+18	-1	0
H_2O	Enhanced	by		1.00E+01
N_2	Enhanced	by		0.00E+00
148	$H+O_2+ N_2=HO_2+ N_2$	6.70E+19	-1.4	0
149	$H+ HO_2= H_2+O_2$	4.30E+13	0	1411
150	$H+ HO_2=2OH$	1.70E+14	0	874
151	$H+ HO_2=O+ H_2O$	3.00E+13	0	1721
152	$O+ HO_2=O_2+OH$	3.30E+13	0	0
153	$OH+ HO_2= H_2O+O_2$	8.90E+16	-1	0
154	$HO_2+ HO_2= H_2O_2+O_2$	4.20E+14	0	11982
Declared duplicate reaction...				
155	$HO_2+ HO_2= H_2O_2+O_2$	1.30E+11	0	-1629
Declared duplicate reaction...				
156	$H_2O_2+M=OH+OH+M$	1.80E+17	0	45500
H_2O	Enhanced	by		5.00E+00
157	$H_2O_2+H= HO_2+ H_2$	1.70E+12	0	3755
158	$H_2O_2+H=OH+ H_2O$	1.00E+13	0	3576
159	$H_2O_2+O=OH+ HO_2$	6.60E+11	0	3974
160	$H_2O_2+OH= H_2O+ HO_2$	7.80E+12	0	1330
Declared duplicate reaction...				
161	$H_2O_2+OH= H_2O+ HO_2$	5.80E+14	0	9560
Declared duplicate reaction...				
162	$IC_8H_{16}= YC_7H_{13}$-Y2+ CH_3	7.05E+66	-14.8	100101
163	$IC_8H_{16}+OH= IC_8H_{15}$-A + H_2O	2.63E+07	1.8	278.2
164	$IC_8H_{16}+OH= JC_8H_{15}$-B + H_2O	6.00E+06	2	-239
165	DMPD13+H= YC_7H_{13}-Y2	1.06E+12	0.5	1230
166	DMPD13+ CH_3= JC_8H_{15}-B	2.92E+03	2.4	8360
167	DMPD13+ CH_3= IC_8H_{15}-A	2.92E+03	2.4	8360
168	DMPD13+OH=DMPD13-E+ H_2O	6.00E+06	2	-239
169	DMPD13-E+ HO_2=DMPD13O+OH	7.00E+12	0	-1000
170	DMPD13O=DMBD13-D+C H_2O	9.37E+18	-1.7	23116
171	DMPD13O=DMPD13HO+H	4.63E+15	-0.8	21016.4
172	DMBD13-D= C_3H_5-T+ C_3H_4-P	9.60E+39	-8.2	42030
173	DMBD13-D+CO=DMPD13CO	1.51E+11	0	4810
174	DMPD13HO+O=DMPD13CO+OH	7.18E+12	0	1389
175	DMPD13HO+OH=DMPD13CO+ H_2O	2.69E+10	0.8	-340
176	$C_3H_3+O_2=CH_2CO+HCO$	3.01E+10	0	2870
Reverse Arrhenius coefficients:		4.88E+11	0	
59470				
177	$JC_8H_{16}= IC_4H_8+ IC_4H_8$	3.96E+27	-5	224293.2
178	$JC_8H_{16}= TC_4H_9+ IC_4H_7$	1.13E+92	-22	459246.3
179	$TC_4H_9=H+ IC_4H_8$	4.65E+46	-9.8	230344.6

Reverse Arrhenius coefficients:	5.89E+44	-9.4	71010.4
180 $IC_4H_8+H= IC_4H_7+ H_2$	3.40E+05	2.5	10421.5
Reverse Arrhenius coefficients:	3.17E+04	2.5	74314.1
181 $IC_4H_8+OH= IC_4H_7+ H_2O$	5.20E+06	2	-1246.2
Reverse Arrhenius coefficients:	5.16E+06	1.9	124958.2
182 $IC_4H_8= IC_4H_7+H$	3.07E+55	-11.5	478002.6
Reverse Arrhenius coefficients:	7.17E+54	-11.7	108773.8
183 $IC_4H_7+ HO_2= IC_4H_7O+OH$	7.00E+12	0	-4182
Reverse Arrhenius coefficients:	3.42E+12	0.1	46963.9
184 $IC_4H_7O=IC_3H_5CHO+H$	5.00E+13	0	121696.2
Reverse Arrhenius coefficients:	6.67E+13	-0.1	76990.6
185 $IC_4H_7+O=IC_3H_5CHO+H$	6.03E+13	0	0
Reverse Arrhenius coefficients:	2.88E+16	-0.5	280779.5
186 $IC_4H_8+O=C\ H_2CO+ CH_3+ CH_3$	3.33E+07	1.8	317.8
187 $IC_4H_8+H= C_3H_6+ CH_3$	5.68E+33	-5.7	83640
Reverse Arrhenius coefficients:	3.06E+26	-4.2	113792.2
188 $IC_4H_7= C_3H_4$-A+ CH_3	1.23E+47	-9.7	310555.3
Reverse Arrhenius coefficients:	1.13E+38	-7.8	99615.2
189 $IC_4H_7O= C_3H_5$-T+C H_2O	2.92E+21	-2.4	147917.3
Reverse Arrhenius coefficients:	1.00E+11	0	52693.2
190 $IC_3H_5CHO+OH=IC_3H_5CO+ H_2O$	2.69E+10	0.8	-1421.9
Reverse Arrhenius coefficients:	4.40E+10	0.8	150886.6
191 $IC_3H_5CHO+ CH_3=IC_3H_5CO$+CH4	3.98E+12	0	36383.4
Reverse Arrhenius coefficients: 3.93E+13 0 127341.9			
192 $IC_3H_5CHO+O=IC_3H_5CO+OH$	7.18E+12	0	5808.8
Reverse Arrhenius coefficients: 1.19E+12 0 85981.9			
193 $IC_3H_5CHO+H=IC_3H_5CO+ H_2$	2.60E+12	0	10873.2
Reverse Arrhenius coefficients: 9.82E+11 0 99824.3			
194 $IC_3H_5CO= C_3H_5$-T+CO	1.28E+20	-1.9	144111.7
Reverse Arrhenius coefficients: 1.51E+11 0 20111.2			
195 C_3H_5-T= C_3H_4-A+H	3.51E+14	-0.4	171002
Reverse Arrhenius coefficients: 8.50E+12 0 8364			
196 C_3H_5-T= C_3H_4-P+H	1.08E+15	-0.6	160965.2
Reverse Arrhenius coefficients: 6.50E+12 0 8364			
197 C_3H_5-T+O_2= CH_3COCH_2+O	3.81E+17	-1.4	23335.6
Reverse Arrhenius coefficients: 2.00E+11 0 73185			
198 C_3H_5-T+O_2=C H_2O+ CH_3CO	3.71E+25	-4	29453.8
Reverse Arrhenius coefficients: 1.87E+27 -4.4 423218.4			
199 $C_2H_2+ CH_3= C_3H_4$-P+H	1.21E+17	-1.2	69755.8
Reverse Arrhenius coefficients: 1.00E+14 0 16728			
200 C_3H_4-P+OH=C3H3+ H_2O	1.00E+07	2	4182
Reverse Arrhenius coefficients: 2.80E+07 1.8 134325.8			
201 C_3H_4-P+H=C3H3+ H_2	2.00E+07	2	20910
Reverse Arrhenius coefficients: 1.30E+07 1.8 87654.7			

202	$CH_3COC H_2$=C H_2CO+ CH_3	1.00E+14	0	129642
Reverse Arrhenius coefficients: 1.00E+11 0 25092				
203	C_3H_4-A+OH=C3H3+ H_2O	1.00E+07	2	4182
Reverse Arrhenius coefficients: 7.00E+06 2 144362.6				
204	C_3H_4-A+H=C3H3+ H_2	2.00E+07	2	20910
Reverse Arrhenius coefficients: 3.24E+06 2 97691.5				
205	CH_3+ HO_2=CH4+O_2	3.60E+12	0	0
Reverse Arrhenius coefficients:		5.18E+15	-0.3	57960
206	CH4+OH= CH_3+ H_2O	1.93E+05	2.4	2106
Reverse Arrhenius coefficients:		4.82E+02	2.9	14860
207	CH_3O_2H= CH_3O+OH	6.31E+14	0	42300
208	CH_2OH+O_2=CH_2O+ HO_2	3.81E+06	2	1641
Reverse Arrhenius coefficients:		1.77E+11	0.7	24180
209	CH_3O2+M= CH_3+O_2+M	4.34E+27	-3.4	30470
Reverse Arrhenius coefficients:		5.44E+25	-3.3	0
210	CH_3O_2+C H_2O= CH_3O_2H+HCO	1.99E+12	0	11670
Reverse Arrhenius coefficients:		8.50E+12	-0.5	7009
211	IC_8H_{16}+CH_3O_2= JC_8H_{15}-B +CH_3O_2H	2.04E+04	2.5	12339.3
212	IC_8H_{16}+ HO_2= JC_8H_{15}-B + H_2O_2	2.04E+04	2.5	12339.3
213	IC_8H_{16}+CH_3O_2= IC_8H_{15}-A +CH_3O_2H	8.10E+04	2.5	16690.8
214	DMPD13+OH=DMPD13-B+ H_2O	3.00E+06	2	-239
215	DMPD13-B+ HO_2=DMPDBO+OH	7.00E+12	0	-1000
216	DMPDBO=M4PD12-D+CH_2O	9.37E+18	-1.7	23116
217	DMPDBO=DMPDBHO+H	4.63E+15	-0.8	21016.4
218	M4PD12-D+ HO_2=M4PD12O+OH	7.00E+12	0	-1000
219	DMPDBHO+OH=DMPDBCO+ H_2O	2.69E+10	0.8	-340
220	M4PD12-D+CO=DMPDBCO	1.51E+11	0	4810
221	M4PD12O=PD12CO+ CH_3	4.86E+14	-0.2	15240
222	PD12CO+OH=PD12CO-E+ H_2O	9.79E+00	3.6	-1128
223	PD12CO-E=C3H3+CH_2CO	1.00E+14	0	31000
224	MPT124+OH=MPT124J+ H_2O	3.00E+06	2	-239
225	MPT124+ CH_3=DMPD13-E	1.50E+11	0	4970
226	C3H3+ C_3H_4-A=MPT124J	8.50E+10	0	11300
227	DMPD13O=DMPD13OHE	1.39E+11	0	15600
228	DMPD13OHE=MPT124+CH_2OH	1.06E+20	-1.9	56180
229	DMBD13-D+O_2=MP1CO-C+O	3.81E+17	-1.4	5580
230	MP1CO-C+ HO_2=MP1OCO+OH	7.00E+12	0	-1000
231	DMBD13-D+O_2=IC_3H_5CHO+ CH_3CO	3.71E+25	-4	7043
232	IC_3H_5CHO+ CH_3CO=MP1OCO	1.00E+11	0	9200
233	IC_8H_{16}+H= JC_8H_{15}-B + H_2	3.79E+05	2.5	2390.1
234	N+NO= N_2+O	2.70E+13	0	355
235	N+O_2=NO+O	9.00E+09	1	6500
236	N+OH=NO+H	3.36E+13	0	385
237	NO_2+ HO_2=HONO+O_2	3.65E+08	1.2	4993

238	CH_3+ NO_2= CH_3O+NO	1.36E+13	0	0
239	CH_3O+ NO_2=C H_2O+HONO	6.02E+12	0	2281
240	CH_3O2+NO= CH_3O+ NO_2	3.00E+12	0	-358
241	C_2H_5+ NO_2= C_2H_5O+NO	1.36E+13	0	0
242	C_2H_5O+ NO_2= CH_3CHO+HONO	4.00E+11	0	0
243	C_2H_5O2+NO= C_2H_5O+ NO_2	3.00E+12	0	-358
244	$C_7H_{15}OO$+NO= $C_7H_{15}O$+ NO_2	4.00E+12	0	-358
245	C_7H_{15}-2+ NO_2= $C_7H_{15}O$+NO	4.00E+13	-0.2	0
246	C_7H_{15}-1+ NO_2= $C_7H_{15}O$+NO	4.00E+13	-0.2	0
247	$AC_8H_{17}O_2$+NO= $AC_8H_{17}O$+ NO_2	1.00E+10	0	0
248	AC_8H_{17}+ NO_2= $AC_8H_{17}O$+NO	1.00E+12	-0.2	0
249	$C_6H_5CH_2OO$ +NO= $C_6H_5CH_2O$+ NO_2	1.03E+14	0	-714
250	$C_6H_5CH_2$+ NO_2= $C_6H_5CH_2O$+NO	1.51E+13	-0.2	0
251	HO_2+NO= NO_2+OH	2.11E+12	0	-479
252	NO+OH(+M)=HONO(+M)	1.10E+14	-0.3	0
Low pressure limit:		2.35E+23	-2.40E+00	0.00E+00
SRI centering:	1.00E+00 0.00E+00 1.00E-18 8.10E-01 0.00E+00			
253	HONO+OH= H_2O+ NO_2	1.26E+10	1	135
254	NO_2+H=NO+OH	1.30E+14	0	360
255	C H_2O+ NO_2=HCO+HONO	8.00E-11	6.7	8300
256	C_6H_5CHO+ NO_2= C_6H_5CO+HONO	8.35E-10	6.7	8300
257	C3H3+N=HCN+C_2H_2	1.00E+13	0	0
258	CH_3+HONO=CH4+ NO_2	8.10E+05	1.9	5504
259	H_2CNO_2+OH=C H_2OH+ NO_2	1.00E+13	0	0
260	CH_3NO_2+OH= H_2CNO_2+ H_2O	1.49E+13	0	5740
261	H_2CNO_2=CH_2O+NO	1.00E+13	0	36000
262	CH_3NO_2(+M)= CH_3+NO_2(+M)	1.80E+16	0	58500
Low pressure limit:		1.30E+17	0.00E+00	4.20E+04
TROE centering:		1.83E-01	1.00E-30	1.00E+30
263	HCCO+NO=HCN+CO_2	1.64E+13	-0.3	-707
264	HCN+O=NCO+H	1.38E+04	2.6	4980
265	NCO+O=NO+CO	2.00E+13	0	0
266	NCO+M=N+CO+M	3.10E+16	-0.5	48000
H_2O	Enhanced	by		1.63E+01
CO	Enhanced	by		1.88E+00
CO2	Enhanced	by		3.75E+00
CH4	Enhanced	by		1.63E+01
C_2H_6	Enhanced	by		1.63E+01
